# A Convolutional Neural Network and Graph Convolutional Network Based Framework for AD Classification

**DOI:** 10.3390/s23041914

**Published:** 2023-02-08

**Authors:** Lan Lin, Min Xiong, Ge Zhang, Wenjie Kang, Shen Sun, Shuicai Wu, Initiative Alzheimer’s Disease Neuroimaging

**Affiliations:** Department of Biomedical Engineering, Faculty of Environment and Life, Beijing University of Technology, Beijing 100124, China

**Keywords:** neuroimaging, Alzheimer’s disease, deep learning, graph convolutional networks

## Abstract

The neuroscience community has developed many convolutional neural networks (CNNs) for the early detection of Alzheimer’s disease (AD). Population graphs are thought of as non-linear structures that capture the relationships between individual subjects represented as nodes, which allows for the simultaneous integration of imaging and non-imaging information as well as individual subjects’ features. Graph convolutional networks (GCNs) generalize convolution operations to accommodate non-Euclidean data and aid in the mining of topological information from the population graph for a disease classification task. However, few studies have examined how GCNs’ input properties affect AD-staging performance. Therefore, we conducted three experiments in this work. Experiment 1 examined how the inclusion of demographic information in the edge-assigning function affects the classification of AD versus cognitive normal (CN). Experiment 2 was designed to examine the effects of adding various neuropsychological tests to the edge-assigning function on the mild cognitive impairment (MCI) classification. Experiment 3 studied the impact of the edge assignment function. The best result was obtained in Experiment 2 on multi-class classification (AD, MCI, and CN). We applied a novel framework for the diagnosis of AD that integrated CNNs and GCNs into a unified network, taking advantage of the excellent feature extraction capabilities of CNNs and population-graph processing capabilities of GCNs. To learn high-level anatomical features, DenseNet was used; a set of population graphs was represented with nodes defined by imaging features and edge weights determined by different combinations of imaging or/and non-imaging information, and the generated graphs were then fed to the GCNs for classification. Both binary classification and multi-class classification showed improved performance, with an accuracy of 91.6% for AD versus CN, 91.2% for AD versus MCI, 96.8% for MCI versus CN, and 89.4% for multi-class classification. The population graph’s imaging features and edge-assigning functions can both significantly affect classification accuracy.

## 1. Introduction

Alzheimer’s disease (AD), a progressive and irreversible neurodegenerative pathology, is manifested by progressive memory impairment and cognitive dysfunction [1]. The disease gradually leads to severe cognitive deterioration and eventual death from complications, which places a tremendous burden on patients, families, caregivers, and society. The relative risk of AD rises dramatically after the age of 65 years, and the number of people affected by the disease is expected to reach 107 million by 2050 [2]. Mild cognitive impairment (MCI) is considered an intermediate state between cognitive normal (CN) and AD. About 40% of MCI patients progress to AD within five years [3]. The average annual conversion rate is about 10–15%. The etiology and pathogenesis of AD remain unclear. Accurate diagnosis of AD at an early stage is critical for timely treatment and possible delays in disease progression. Grey matter atrophy is associated with cognitive decline in chronological ageing, MCI, and AD dementia. The magnetic resonance imaging (MRI) technique has significantly increased our understanding of brain atrophy in AD and has been successful in examining the differences between AD patients and healthy controls. However, in routine clinical practice, the interpretation of MRI scans is largely based on the clinician’s experience and intuition. With the rapid development of neuroimaging analysis, automatic classification based on MRI scans may be useful in improving diagnostic accuracy and reducing differences among clinicians, leading to reduced medical costs.

Machine learning is widely used in medical imaging because it facilitates the identification of patterns in large datasets. Numerous algorithms have been proposed to extract features from MRI and make individual predictions. Deep learning methods, such as convolutional neural networks (CNNs) [4], have achieved favorable and competitive performance compared to the classical machine-learning algorithms for high-dimensional MRI images, as they are able to automatically extract features in different abstraction levels. Kang et al. [5] introduced a multi-slice and multi-model ensemble learning approach based on a 2D CNN for learning the various features from local brain images, which were then integrated for the final classification. It had a high discrimination power for AD versus CN, with an accuracy of 90.36%, and an accuracy of 77.19%, and 72.36% for AD versus MCI, and MCI versus CN, respectively. Liu et al. [6] used a UNet in conjunction with 3D densely connected convolutional networks (3D DenseNets) to jointly perform hippocampal segmentation and AD classification, and achieved accuracy values of 88.9% and 76.2% when classifying AD versus CN, and MCI versus CN, respectively. CNNs are suitable for extracting features from Euclidean neuroimaging data, and the convolutional layers work as filters. However, CNN-based methods cannot properly capture the complex non-grid structural and functional representations of the brain. Graph convolutional networks (GCNs) generalize convolution operations to non-Euclidean data, and some researchers have started to use them to analyze brain networks. Meszlenyi et al. [7] modeled functional connections between brain regions as graphs and used a two-layer GCN for MCI classification. When classifying MCI versus CN, the accuracy was 71.9%. Song et al. [8] used diffusion tensor imaging to build structural networks. BrainNetCNN, which is composed of edge-to-edge, edge-to-node, and node-to-graph convolutional filters, was utilized for classification tasks among CN, early MCI, late MCI, and AD.

**Figure 1:**
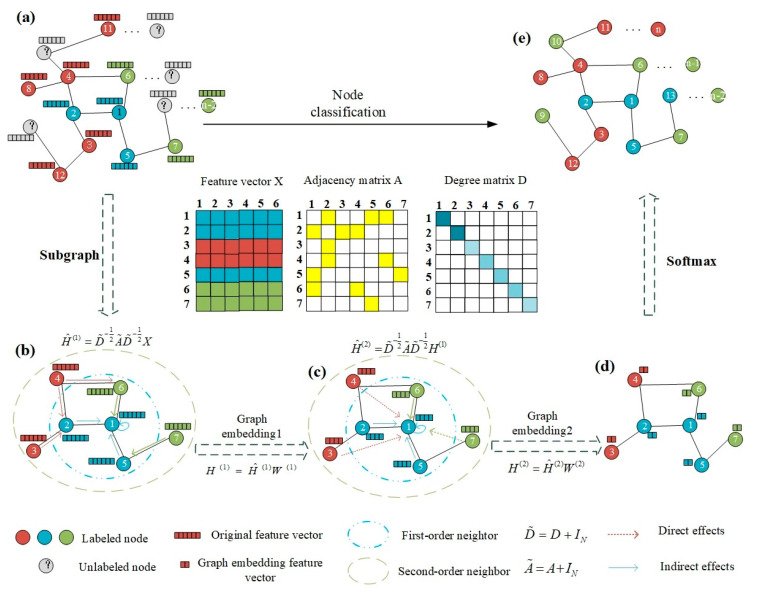
Process of a node classification task. (**a**) Input population graph (**b**) the first graph convolution (**c**) the second graph convolution (**d**) output layer (**e**) Output population graph.

The aforementioned GCN-based studies, however, did not incorporate the similarities among the individuals in the graph architecture. The use of a population graph is beneficial for assessing relationships among subjects. The population data can be viewed as a graph, with the individual samples in the cohort acting as nodes and phenotypic data and/or pairwise similarities of imaging features between subjects acting as edges. Modeling the population data with a graph transforms the AD classification problem into a node classification problem. As shown in Figure 1, the task is to predict the diagnostic label of an unseen subject drawn from a test dataset (subjects with gray nodes). The graph is represented as G (V, E, X), with N nodes and M edges, where V is the set of nodes and each subject corresponds to a node in the graph. The edges E represent relationships between the nodes. A is the adjacency matrix with N × N elements, D is the degree matrix of A, IN is the identity matrix, and X indicates the node feature matrix. Every node vi has a corresponding feature vector xi and a true label ci ∈ C, where C is the set of classes. GCN is used to learn the parametric function F with the inputs X, A, and D. The graph convolutional layer comprises two steps. First, it fuses each node’s information with that of its neighbors based on their edge connections, and then it constructs node embeddings based on the updated features using a fully connected layer.

Consequently, considerable effort has been devoted to developing GCN models with a population graph structure. Kazi et al. [9] constructed multiple population graphs with various biomarkers (MR, PET imaging, cognitive tests, and CSF biomarkers) as node features and age, gender, ApoE genotype, and other variables as edges. The features extracted by each GCN were merged for the final classification. The self-attention mechanism was used in GCN to improve the quality of information aggregation under the GCN framework. The classification accuracy of AD, MCI, and CN was 76%. Researchers [10] made use of a dynamic high-order brain functional connectivity network constructed from resting state functional magnetic resonance imaging time series. The characteristics of the brain’s functional connectivity network were combined with gender and age information to build a population graph. InceptionGCN, which uses multiple scale convolution kernels, was introduced to improve the model’s performance. For the task of comparing early MCI with late MCI, classification showed 79.2% accuracy. Jiang et al. [11] proposed a hierarchical GCN framework with two major components: a graph-level GCN and a node-level GCN. Individual brain functional connectivity network features were extracted using the graph-level GCN, and those features were combined with non-imaging complementary data to create a population graph. The node-level GCN was used for graph embedding learning and classification. The model obtained an accuracy of 78.5% for AD versus MCI.

Most of the aforementioned studies focused on developing better GCN architectures and accordingly proposed various GCN variants. The function of GCN in a population graph is to build node embedding by fusing the features of the nodes in the graph structure using the relationships with the immediate neighbors. GCN can be viewed as a special type of Laplacian smoothing for node features over graph structures [12]. An over-smoothing problem [13,14] caused by too many layers of aggregation/propagation steps, produces indistinguishable representations of nodes, degrades the model’s performance, and increases computational complexity. Thus, GCN models are commonly constrained to a shallow architecture, but shallow embedding may not sufficiently propagate node features for fusing heterogeneous information. Furthermore, the features are fused by considering the population graph’s topological structure. Because the learning range of node embedding is affected by the edge-assigning function, distinct feature vectors are created. Few studies have looked into how the input properties of GCN (edges and features) influence AD staging performance. This motivated us to investigate the impact of feature importance and node interactions on GCN-based AD staging using population graphs.

This study was designed to investigate how the input characteristics of GCNs affect the performance of AD staging. The research objectives were to answer the following research questions: (1) Does including demographic information in the edge-assigning function lead to better classification performance when classifying AD versus CN? (2) How does adding various neuropsychological tests to the edge-assigning function affect the classification of MCI? (3) Does the edge assignment function that performs best in MCI classification also perform well in multiclass classification? To achieve this objective, we proposed a novel framework by leveraging the superior feature extraction capabilities of CNNs and the population-graph processing capabilities of GCNs. DenseNet was used to learn high-level anatomical features. A set of population graphs with nodes defined by imaging features and edge weights determined by different combinations of imaging or/and non-imaging information were fed to GCNs for classification.

The remainder of the paper is organized as follows: Section 2 covers the data source and data preprocessing, the overall framework of the experiment, the creation of the population graph, the learning principle of GCN, and the evaluation index of the model. Section 3 introduces the specific settings and results of the experiment. Section 4 provides the experimental results, and Section 5 offers a summary of our findings and some concluding comments.

## 2. Materials and Methods

### 2.1. Participant

The data employed in the preparation of this article were obtained from the Alzheimer’s Disease Neuroimaging Initiative (ADNI) database (http://adni.loni.usc.edu/, accessed on 15 February 2020). The ADNI was launched in 2003 as a public–private partnership, led by Principal Investigator Michael W. Weiner, MD. The primary goal of ADNI has been to test whether MRI, PET, other biological markers, and clinical and neuropsychological assessment can be combined to measure the progression of MCI and AD. All ADNI participants provided written informed consent, and the institutional review board of each ADNI site approved study protocols.

On 1.5 Tesla MRI scanners from Siemens (Siemens, Erlangen, Germany), Philips (Philips, Best, The Netherlands), and General Electric Health-care (General Electric Health Care, Waukesha, WI, USA), high-resolution T1-weighted structural MRI (sMRI) data at baseline were collected at multiple ADNI sites using the standard ADNI Phase 1 (ADNI-1) MRI protocol. A sagittal 3D MP-RAGE sequence was used to scan each subject, with the following acquisition parameters: inversion time/repetition time: 1000/2400 ms; flip angle: 8; 24 cm field of view; 192 × 192 × 166 acquisition matrix, and a voxel size of 1.25 × 1.25 × 1.2 mm^3^. In plane, zero-filled reconstruction yielded a 256 × 256 matrix for a reconstructed voxel size of 0.9375 × 0.9375 × 1.2 mm^3^. In order to assure uniformity among scans obtained at different sites, images were calibrated using phantom-based geometric corrections. Additional image corrections were also applied, to adjust for scanner- and session-specific calibration errors. In addition to the original uncorrected image files, images with all these corrections already applied (GradWarp, B1, phantom scaling, and N3) are available to the general scientific community (at www.loni.ucla.edu/ADNI, accessed on 15 February 2020). The samples included in the ADNI-1 cohort were diagnosed with 3 clinical statuses (CN, MCI, and AD), including 187 AD patients, 382 MCI patients, and 229 CNs at baseline. The neuropsychological assessments used in this study could be divided into global cognitive screening tests, the Functional Assessment Questionnaire (FAQ), and ADNI composite scores. Global tests consist of the Mini-Mental State Examination (MMSE), sum-of-box assessments of clinical dementia (CDR-SB), the 11-item AD Assessment Scale-Cognitive (ADAS-Cog11) or expanded to 13 items (ADAS-Cog13). The ADNI composite scores include four sub-domains: memory, executive function, language, and visuospatial. Gibbons et al. derived the composite scores for memory (ADNI-MEM) and executive function (ADNI-EF) from the ADNI neuropsychological battery using item response theory [15,16] and Choi et al. designed the composite scores for language (ADNI-LAN) and visuospatial abilities (ADNI-VS) using similar methods [17]. The demographic details and neuropsychological assessment [18] results for the three groups are provided in Table 1. The dataset was randomly split into 70% training, 10% validation, and 20% test sets. The training set was used to train the algorithm, the validation set was used to find the optimal combination of hyper-parameters, and the test set was used to evaluate the model.

### 2.2. Image Preprocessing

Brain imaging data were converted from DICOM images to Neuroimaging Informatics Technology Initiative (NIFTI) files using dcm2nii from the MRIcron package (http://people.cas.sc.edu/rorden/mricron/index.html, accessed on 20 December 2022). Images were manually reoriented with the coordinate system’s origin set to the anterior commissure. Voxel-based morphometry analysis was performed on the structural imaging data with the Computational Anatomy Toolbox (CAT12) toolbox (http://www.neuro.uni-jena.de/cat/, accessed on 20 December 2022), an extended toolbox of SPM12 [15], with default settings. The preprocessing pipeline included realignment, skull stripping, segmentation by tissue type (i.e., gray matter and white matter), and finally, the segmented gray matter images were non-linearly warped to the standard Montreal Neurological Institute (MNI) template [19], modulated to account for volume changes. Modulated and warped 3D gray matter density maps (GMDMs) were smoothed using a 2-mm full width at half maximum Gaussian kernel. The GMDMs had a dimensionality of 121 × 145 × 121 in the voxel space (a voxel size of 1.5 × 1.5 × 1.5 mm^7^). The GMDMs were further re-sampled to an isotropic voxel size of 3 × 3 × 3 mm^3^ to provide an image dimension of 64 × 64 × 64 for an efficient computation.

### 2.3. Densenet for Gmdms Feature Learning

DenseNet, an extension of the ResNet architecture, was proposed by Huang et al. [20]. To maximize the information flow through layers, the DenseNet architecture uses a simple connectivity pattern in which each layer in a dense block obtains the feature maps from all previous layers and passes its own feature maps to all subsequent layers. With this architecture, DenseNet has several advantages, including preventing over-fitting and degradation phenomena, improving the efficiency of feature propagation, retaining the efficiency of feature reuse, and substantially reducing the model’s size.

**Figure 2:**
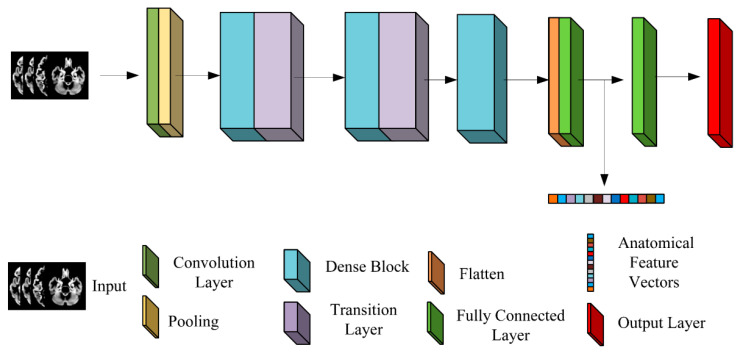
Illustration of the overall architecture of the 3D Densenet model.

The GMDMs were used as inputs for the model. DenseNet, trained from scratch, was used to investigate a binary problem (AD versus CN). To generate the optimal model for AD versus CN, we empirically tuned DenseNet’s hyper-parameters using a grid-search technique, such as the learning rate (1 × 10^−6^–1 × 10^−2^), the number of dense blocks (2–5), the growth rate (8–24), the compression rate (0.2–0.8), and the batch size (32–128), according to the validation results. While changing the values of the hyper-parameter, mean values for accuracy (ACC) were calculated for each value of the hyper-parameter. In the cost function calculation, balanced class weights were used to ensure that classes were weighted inversely proportional to their frequency in the training set. A schematic of the optimized 3D DenseNet architecture is shown in Figure 2. It consisted of a 3 × 3 × 3 convolutional layer, followed by three dense blocks, and a transition layer in between. The output of the last dense block is flattened, followed by two fully connected layers with 512 units and 256 units, respectively, and finally connected to the output layer. Each dense block has three repeating units: each repeating unit has one bottleneck 1 × 1 × 1 convolutional layer with 48 channels, followed by a 3 × 3 × 3 convolutional layer with 12 channels. The loss function was binary cross-entropy. The learned hyper-parameters are shown below; the learning rate, growth rate, compression rate, and batch size were set at 0.0001, 12, 0.5, and 64, respectively. A transfer learning strategy was applied to this optimized DenseNet architecture to initialize the training of the CNNs for two binary (AD versus MCI and MCI versus CN) and one multiple-class classification problem (CN, MCI, and AD). This was done primarily because of the fact that these four tasks are highly associated, and the latter jobs are substantially more demanding. Training was performed using Adam optimization. The model is implemented in Keras using Tensorflow as the backend and trained on an NVIDIA GTX 3090 GPU with 24 GB of RAM. After training, the anatomical features of the GMDMs were extracted from the first fully connected layer. The CNN model was trained for a maximum of 200 epochs, and early-stopped after 30 epochs of the validation loss not improving.

### 2.4. Population Graph Construction

To consider the correlations among the subjects in a cohort, the population is regarded as a graph. Individual subjects are represented by the nodes of the population graph, which include compact anatomical feature vectors taken from 3D DenseNet, while the edges encode pairwise phenotypic similarities based on non-imaging and/or imaging data. The population graph is constructed using the set of CN and patients with MCI and AD. The subjects from the dataset are represented by the graph nodes, and similarities between the nodes’ characteristics, such as demographic, imaging, and/or neuropsychological features, are treated as edges connecting the nodes. A population graph is constructed based on two important elements: (a) the node feature vector assigned to each node, and (b) the weighted adjacency matrix. More explicitly, we built an undirected weighted graph G (V, E, X) in which the set of nodes V = {v_1_,⋯,v_n_} corresponds to a set of subjects. Each node v_i_ contains a 512-dimensional feature vector x_i_ described in Section 2.3. The feature matrix X∈R^n×512^ consists of stacked feature vectors of n nodes in the graph. The weighted adjacency matrix A is composed of a set of edges E ⊆ V × V, which correspond to links between the nodes, where an edge-assigning function assigns weight *S*(*i*, *j*) to each edge. However, constructing a population graph is not a straightforward task, as there are multiple edge-assigning functions that map the data to the graph structure. Edge-assigning function is critical for capturing the underlying structure of a graph and explaining the similarities between the feature vectors. We computed the similarity between the pair of anatomical feature vectors *x_i_* and *x_j_* of nodes *i* and *j*. The similarity index was denoted as $S_{img}(i,j)$.

(1) $$S_{img}\left(i,j\right)=\frac{x_{i}\cdot x_{j}}{\left\|x_{i}\|\|x_{j}\right\|}$$

A similarity function $S_{nimg}(i,j)$ is defined as a Kronecker delta function if the non-imaging feature is categorical (e.g., subject’s gender). The function is specified as a unit-step function with regard to a threshold β if the non-imaging feature is quantitative (e.g., subject’s age).

(2) $$S_{nimg}\left(i,j\right)={\{}_{0\;otherwise}^{1\;if{\;n}_{i}=n_{j}\;}$$

(3) $$S_{nimg}\left(i,j\right)={\{}_{0\;otherwise}^{1\;if{\;n}_{i}-n_{j}<\beta }$$

In the equations above, $n_{i}$ and $n_{j}$ are the values of the non-imaging features for nodes *i* and *j*.

The combined similarity index is defined by the equation below.

(4) $$S_{\mathrm{com}}\left(P,i,j\right)=S_{img}\left(i,j\right)\sum_{p=1}^{P}S_{nimg}\left(i,j\right)$$

where *P* is the number of non-imaging features that has been used to generate edges. Equation (4) states that $S_{\mathrm{com}}$ increases when there is a high degree of similarity between two subjects’ imaging feature vectors and/or their non-imaging measures. Non-imaging features and imaging features are incorporated.

For clarity, we categorized the resulting graphs into three groups based on their edge-assigning functions:

Baseline graphs: Graphs were constructed using the similarity between imaging feature vectors described in Section 2.3.

Non-imaging graphs: Graphs were constructed using the relationships between non-imaging features.

Combined graphs: Graphs that were constructed using a combination of non-imaging and imaging features.

To examine how the construction of the population graph (edges and features), especially the edge-assigning function, influences AD staging performance, three experiments were implemented in this study. Experiment I was designed to explore the implications of incorporating demographic information in the edge-assigning function on the classification of AD versus CN. Experiment II was designed to investigate the impact of adding various neuropsychological tests to the edge-assigning function on MCI classification. Experiment III aims to investigate the possibility of using the edge-assigning function, which produced the best outcomes in Experiment II, to perform well on multi-class classification.

- Experiment 1: Demographic information-based population graph for AD versus CN classification

Individuals with AD usually demonstrate a high level of heterogeneity [21]. Some atrophic areas affected by one AD subtype may be preserved by another [22,23]. As a result, imaging features and AD risk factors should be combined in the diagnosis of AD. One of the biggest risk factors for AD is aging; more than 13% of people aged 65 and up and 43% of people aged 85 and up have been diagnosed with AD [24]. Genetic factors also play a role. Apolipoprotein E (ApoE) is a well-known risk factor for late-onset AD [25,26]. Female birth sex has been linked to an increased risk of developing AD, and two-thirds of older adults with AD are women [27,28]. Therefore, non-imaging information such as age, gender, and ApoE genotype was used to calculate the similarity of the nodes in this investigation. Based on all possible combinations, seven population graphs were created. A grid search with validation was used to determine the threshold of age;

- Experiment 2: Neuropsychological assessments-based on the population graph for MCI classification

Of note is the fact that distinguishing MCI patients from CN subjects or AD patients based on neuroimaging data is more difficult than distinguishing between AD and CN, and the results of the former are always less accurate [29]. The criteria for clinically categorizing ADNI-1′s subjects into different disease groups were summarized as follows [30]: (a) CN subjects with normal cognition and memory, MMSE between 24–30, CDR = 0, non-depressed (b) MCI patients with verified memory complaint, MMSE between 24–30, CDR = 0.5, have objective memory loss measured by education adjusted scores on Wechsler Memory Scale Logical Memory II, absence of significant levels of impairment in other cognitive domains, essentially preserved activities of daily living, or (c) probable AD with validated memory complaint, MMSE in the range of 20–26 and CDR ≥ 0.5, and met NINCDS/ADRDA criteria for probable AD. Because neuropsychological tests, particularly the MMSE and CDR, were employed as major criteria in categorizing participants, they could provide complementary information for MCI classification. Non-imaging information from nine neuropsychological assessments was utilized to compute the similarity of the nodes in the population graph, and 18 population graphs were created, nine with a non-imaging similarity index as edges and nine with a combined similarity index as edges. The optimal threshold *β* of each neuropsychological assessment for each task was determined through an exhaustive grid search with validation;

- Experiment 3: Population graph for multi-class classification

Most AD and MCI research normally simplifies the classification problem to a set of binary classification tasks, such as AD versus CN and/or MCI versus CN. However, AD staging should be naturally modeled as a multi-class classification problem, necessitating the examination of the entire AD spectrum. The classification of AD, CN, and MCI is difficult because a multi-class model has more interference than a two-class model. In the current study, the edge-assigning function that achieved the best result in the MCI classification was used for multi-class classification.

### 2.5. GCN

After constructing the population graph represented in Section 2.4, we learn the GCNs to predict the target labels. Various GCN frameworks have been proposed, and one of the most seminal examples was proposed by Kipf and Welling [31] in 2016. The GCN model architecture is composed of stacked layers of graph convolution, with each layer’s propagation rule described as:

(5) $$\hat{D}=\;D+\;I$$

(6) $$\hat{A}=\;A+\;I$$

(7) $$H^{\left(l+1\right)}=f\left(H^{l},A\right)=\sigma ({\hat{D}}^{-1/2}\hat{A}{\hat{D}}^{-1/2}H^{\left(l\right)}W^{\left(l\right)})$$

where *D* and *A* are the degree matrix and adjacency matrix, respectively, *I* is the identity matrix, and $\hat{D}$ is the diagonal node degree matrix of $\hat{A}$, *W*^(*l*)^ are the network parameters of the lth layer to be learned, *H*^(*l*+1)^ are the node embeddings, *H*^(*l*)^ are generated from the previous message-passing step, and *f* represents a non-linear activation function. ${\hat{D}}^{-1/2}\hat{A}{\hat{D}}^{-1/2}$ is intended to add a self-connection to each node and keep the scale of the feature vectors. During training, the vertices connected with high edge weights become more similar as they pass through multiple layers.

**Figure 3:**
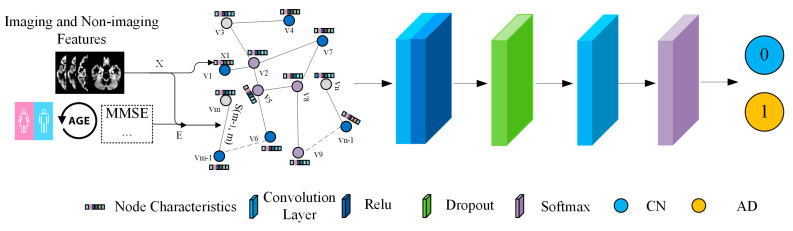
Illustration of the overall architecture of the GCN model for binary classification between the AD and CN classes.

From the perspective of message passing, two steps were performed: (1) producing an intermediate representation by aggregating information for a node from its neighbors; and (2) transforming the aggregated representation with a linear transformation parameterized by W shared by all nodes, which was followed by non-linearity activation. In the current study, we built a GCN model (Figure 3) by stacking two graph convolutional layers with the adjacency and node feature matrices as inputs, and the activation function of the first convolutional layer is ReLU. It’s worth noting that the first graph convolutional layer has 32 neurons and that the second graph convolutional layer has two neurons (for binary classification) or three neurons (for three-class classification), followed by a soft-max activation function. The loss function is defined by the difference between the predicted label and the actual label, where a cross-entropy loss function is used in our implementation. For GCN, we adopted code from the GCN in PyTorch GitHub repository (https://github.com/tkipf/pygcn (accessed on 20 December 2022)). The model was trained using a grid-search technique in order to find the optimal combination of hyper-parameters (learning rate and dropout ratio) for this architecture. The range of the hyper-parameter values was (1 × 10^−6^–1 × 10^−2^ for learning rate and 0.3–0.8 for dropout ratio). The training was conducted using the Adam optimizer implemented in PyTorch. The optimal learning rate was 0.001, 0.0001, and 0.0001 for Experiments I, II, and III, respectively, and dropout was 0.5. The maximum epoch was set at 500 for all the tasks, with a criterion to stop training if the accuracy on the validation set did not improve after 20 epochs. During the training, we use the entire set of data, including labeled training and unlabeled test samples, to construct the whole population graph. The GCNs are trained to minimize the cross-entropy loss for all training samples. After training the GCNs, the model will output a prediction for each test sample.

### 2.6. Evaluation Metrics

In order to evaluate the performance of the proposed model, three common metrics were used. The accuracy (ACC) gives an overview of the quality of the predictions. The precision (PRE) shows the ratio of the correct predictions out of all the predictions, the recall (REC) is the percentage of how many total positive cases there are in all positive samples, the F1 score is a harmonic mean of ACC and PRE, and the Matthews correlation coefficient (MCC) considers all elements of the confusion matrix, providing a better view of the performance of classifiers. The calculation of those metrics is based on Equations (8)–(12), respectively.

(8) $$\mathrm{ACC}=\frac{\mathrm{TP}+\mathrm{TN}}{\mathrm{TP}+\mathrm{TN}+\mathrm{FP}+\mathrm{FN}}$$

(9) $$\mathrm{PRE}=\frac{\mathrm{TP}}{\mathrm{TP}+\mathrm{FP}}$$

(10) $$\mathrm{REC}=\frac{\mathrm{TP}}{\mathrm{TP}+\mathrm{FN}}$$

(11) $$F1=\frac{2\times \mathrm{PRE}\times \mathrm{REC}}{\mathrm{PRE}+\mathrm{REC}}$$

(12) $$\mathrm{MCC}=\frac{\mathrm{TP}\times \mathrm{TN}-\mathrm{FP}\times \mathrm{FN}}{\sqrt{\left(\mathrm{TP}+\mathrm{FN}\right)\times \left(\mathrm{TP}+\mathrm{FP}\right)\times \left(\mathrm{TN}+\mathrm{FP}\right)\times \left(\mathrm{TN}+\mathrm{FN}\right)}}$$

where TP, TN, FP, and FN are the abbreviations for true positive, true negative, false positive, and false negative, respectively.

## 3. Results

3D DenseNet achieves a relatively good performance for AD versus CN (ACC scores of 84.3%, PRE scores of 83.3%, and REC scores of 81.1%). But the performance is lowered for AD versus MCI and MCI versus CN, showing ACC scores of 70.7% and 71.9%, PRE scores of 74.1% and 58.1%, and REC scores of 81.1% and 48.6%, respectively. The anatomical features extracted from the first fully connected layer were used as node features for graph learning.

### 3.1. Experiment 1

There is no simple way to create a population representation of the data, as the data needs to be mapped onto the graph structure. The optimal graph structure would be one that allows the clustering of AD and CN to be easily separable from each other. The goal of experiment I was to explore the effects of incorporating demographic information into the edge-assigning function on the classification of AD versus CN. Non-imaging complementary data (age, gender, and ApoE genotype) were used to estimate subjects’ similarity. The validation set and grid search were used to optimize the threshold, which yielded an optimal threshold of 2 for age. The results are provided in Table 2. For instance, we investigated whether a non-imaging feature would improve performance when used alone. In the population graph with only imaging features in the edge-assigning function, we observed that the performance did not change much. Adding the ApoE4 genotype to the graph’s edge-assigning function increased the performance, allowing all the graph structures with ApoE to beat the performance of the models without ApoE. The best performance was obtained when $S_{\mathrm{com}}$ was used with age, gender, and ApoE in the edge-assigning function, which showed a 91.6% accuracy. The model’s good performance indicated that both the features and the structure of the population graph (i.e., using graph edges to combine demographic information and imaging data) contained useful information for classification.

### 3.2. Experiment 2

To investigate the effect of adding various neuropsychological tests to the edge-assigning function on MCI classification, Experiment 2 was implemented. The optimal values of the threshold parameters were determined using a grid search approach on the validation set. Table 3 shows the threshold values for AD versus MCI and MCI versus CN.

For a fair comparison, all GCN models were made to employ the same parameter configuration and training method as Experiment II, except for the edge-assigning function. The default graph is based on $S_{img}$, similarity between anatomical features. Nine population graphs were created based on non-imaging neuropsychological assessment scores, with $S_{nimg}$ as the edge-assigning function. The other nine population graphs were constructed with $S_{\mathrm{com}}$ as the edge-assigning function. The results for AD versus MCI are reported in Table 4. The classification performance of the GCN based on the default graph was relatively low. We observed a large variation between the graph structures, with a 23.7% difference in accuracy between the best- and worst-performing graphs ($S_{nimg}\;\mathrm{or}\;S_{\mathrm{com}}$). The best-performing graph was the one ($S_{nimg}$) that used the similarity of CDR-SB in the edge-assigning function. With regard to REC, the best-performing graph shows a relatively higher improvement (27.1%) than the default graph. It is reasonable to deduce that the improved REC enhanced by the edge-assigning function has a more pronounced effect on a more difficult classification task (i.e., AD vs. MCI).

The results for the MCI versus CN are shown in Table 5. The default graph is also based on $S_{img}$. We observed a large variation between the graph structures, with a 34.2% difference in accuracy between the best- and worst-performing graphs. The best-performing graph was also the one that used the similarity of CDR-SB in the edge-assigning function.

### 3.3. Experiment 3

**Figure 4:**
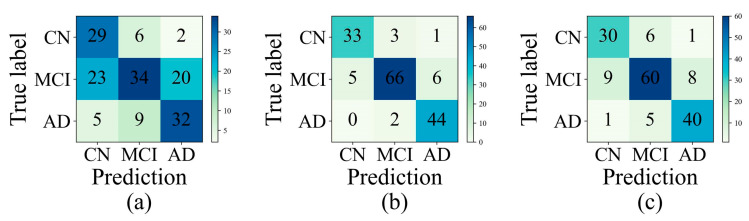
Confusion matrices of three-group (AD, MCI, and CN) classification on the test data: (**a**) the default graph with $S_{img}$ as the edge-assigning function; (**b**) the graphs with $S_{nimg}$ as the edge-assigning function; and (**c**) the graphs with $S_{\mathrm{com}}$ as the edge-assigning function.

After determining the best-performing edge-assigning function (CDR-SB) in Experiment 2, we designed Experiment 3 to test whether CDR-SB would also work well on multi-class classification. The confusion matrix was used as a tool to assess model classification performance on the test data. Figure 4 shows the confusion matrices that give a visual representation of how well the predictions match the actual diagnoses. The darker diagonal cells can be seen in all of the plotted confusion matrices, indicating a high level of accuracy. Model misclassifications are indicated by the off-diagonal elements with light shades. There are two common misclassifications: predicting a CN diagnosis when a patient actually has MCI, and predicting an AD diagnosis when a patient actually has MCI, highlighting the difficulty of distinguishing MCI from CN or AD. The default graph with as the edge-assigning function is not sensitive (44.2%) for identifying MCI patients, but the graphs with as the edge-assigning function and the graphs with as the edge-assigning function have relative high sensitivity (85.7% and 77.9%, respectively). The default graph achieved 59.4% accuracy for the multi-class classification based on $S_{img}$. The graphs with $S_{nimg}$ and $S_{\mathrm{com}}$ as the edge-assigning function, respectively, achieved 89.4% and 81.3% accuracy. Based on the results, we conclude that the population graph with CDR added in the edge-assigning function can significantly outperform the population graph without it, providing a performance gain in accuracy between 21.9% and 30%.

### 3.4. Graph Features versus Vector Features

**Figure 5:**
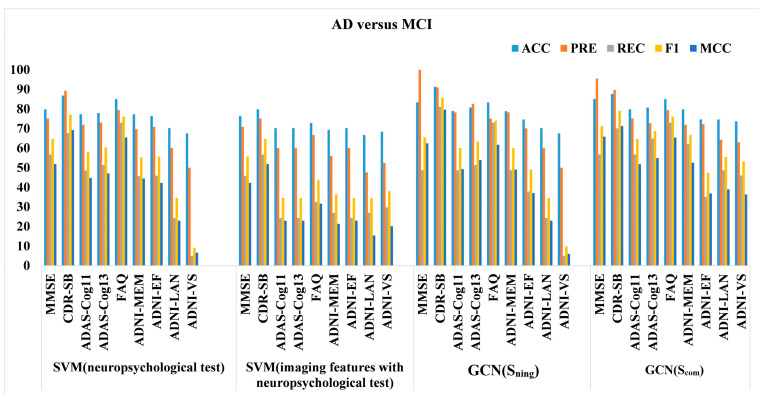
Performance comparison using graph features or vector features on the test data for AD versus MCI task.

**Figure 6:**
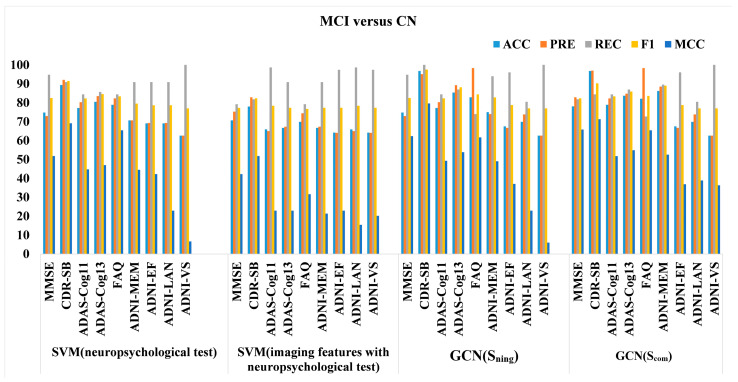
Performance comparison using graph features or vector features on the test data for MCI versus CN task.

Apart from investigating how the edges of the population graph impact the classification performance, we further investigated whether the graph feature structure would allow us to extract an improved feature representation after the graph convolution compared to the vector feature. It is implemented by comparing the GCN results (both using a neuropsychological test score as the edge-assigning function or using the combined features as the edge-assigning function) to those of support vector machine (SVM) with a neuropsychological test score, and SVM with combined features. For SVM with a neuropsychological test score, we used a neuropsychological test score as input. For SVM with combined features, we used the same features as we did for GCN implementation. As shown in Figure 5 and Figure 6, in most cases, the models with a graph feature structure as the input outperformed those with a vector feature structure as the input. Regarding classification accuracy, the GCNs with neuropsychological assessment scores in their edge-assigning function performed better in the first seven and six comparisons for AD versus MCI, and MCI versus CN, respectively.

## 4. Discussion

In this work, we demonstrate the value of the GNN-based graph classification framework along with the 3D DenseNet features for accurate AD categorization. First, hidden feature representations from the anatomical GMDM data were extracted using 3D DenseNet. A set of population graphs was then represented graphically, with nodes defined by imaging features and edge weights indicated by different combinations of imaging/non-imaging information. Finally, GCNs were used to learn the graph structures. Our findings confirmed our initial hypothesis that imaging features and pairwise information are very important in the categorization process.

Understanding heterogeneity in AD can greatly contribute to clinical trial designs and treatment. A structured population graph is an effective way to address heterogeneity and understand the relationships between subjects. Simply put, a graph is a non-linear data structure that represents relationships between subjects and can be used as a powerful abstraction to encode an intrinsic structure. Examining the related neighbors in a graph can reveal important details about a subject’s local relationship. Detecting clusters of AD patients in a population graph necessitates an examination of the global structure, which is composed of the local relationships of many individual nodes interacting with each other. GCNs are designed to work on the relationships between subjects; they are capable of finding structures and revealing patterns in connected subjects. The traditional machine learning method analyzes complementary information in isolation and ignores neighborhood relationships and complicated network structures. The population graph divides complementary information into features and topology, which yields deeper insights into the underlying information of the data. The imaging features are now a set of embedding features, and the relationships between the subjects are encoded in the topology; this structure improves the model’s predictability. Based on the graph structure, the GCN could create new, more meaningful graph embeddings and outperform traditional machine learning methods even when the same information was given as input.

When compared to typical machine learning methods, GCNs are more effective at learning representations of non-Euclidean graph data. The main idea is to perform a convolutional operation on the graph, which enables the network to achieve a new representation of a given node by propagating graph topological information across the neighborhood of each node, which naturally fuses both the graph structure and node features between nodes. Different features or feature interactions inherently have various influences on the convolutional layers. Because message propagation techniques are a type of Laplacian smoothing, learning a node representation by recursively aggregating its neighbors’ information could result in node representations that are indistinguishable. The representations of all nodes tend to converge on the same value as the number of layers grows, leading to over-smoothing. As a result, GCN architectures are typically shallow. GCNs, which focus on obtaining the low-dimensional embedding of the constructed graph, lack CNNs’ powerful feature extraction ability. The cascading architecture of a CNN makes it simple to transfer from low-level common features to high-level complex features to achieve great expressive capability. A key contribution of this research was the use of 3D DenseNet’s high-level features as node descriptors. Unlike other CNNs, which only use the last high-level feature maps, 3D DenseNet applies feature reuse to maximize the network’s capability. The model is more effective when both high-level complexity and low-level common feature maps are used. Because DenseNet’s channel is narrow, it performs well with a significantly reduced number of network parameters. The use of 3D DenseNet to encode graph characteristics can produce better results than the use of raw anatomical features.

Due to complex graph structures, learning about graphs is challenging in that effective ways to incorporate different sources of information into edges must be found. Kipf and Welling [31] used a re-normalized first-order adjacency matrix to approximate the polynomials and combined graph node features and graph topological structural information for classification purposes. In the AD versus CN classification task, AD risk factors were used to calculate the similarity of the nodes. The results showed that using ApoE genotype or gender in the edge-assigning function improved the model’s performance. The graph with age, ApoE genotype, and gender information achieved the best results. ApoE is the primary carrier of cholesterol in the central nervous system, and the ApoE genotype is a strong risk determinant for developing AD. AD patients with at least one ApoE e4 allele accounted for over 60% [32] of the patients. Sex-based prevalence of AD was also well documented, with over 60% of the patients being female [33]. Ghebremedhin et al. [34] found an association between ApoE e4 and AD-related neurofibrillary tangle formation and senile plaques, which were differentially modified by age and gender. Moreover, Riedel et al. [35] found complex interactions between age, ApoE genotype, and gender and believed that the precision medicine approach for AD should be based on the convergence of such three risk factors. These findings explain why the combined similarity index achieved the best results in AD versus CN classification.

MCI is the transitional state between AD and CN, and its most common manifestations are memory deficits. Various neuropsychological assessments were performed on the subjects of the ADNI cohort. Because of these neuropsychological assessments’ quantitative measurements, thresholds are needed for edge-assigning. Different thresholds determine the corresponding levels of the topological structure in the population network. In other words, a larger threshold value often preserves fewer connections and thus has sparser connections. More neighborhood information promotes better node embedding learning. Nevertheless, too much neighborhood information inevitably leads to over-smoothing. If the threshold is too large, the nodes of the population network will not obtain sufficient information from the correlated nodes. Although an exhaustive grid search was used to determine the optimal threshold of each neuropsychological assessment for each task, the determined threshold could be partially clinically important differences in clinical outcome assessments revealed by Andrews et al. [36], who discovered that a 1- to 3-point decrease in MMSE, a 1- to 2-point increase in the CDR-SB, and a 3- to 5-point increase in the FAQ were indicative of a meaningful decline. In the current study, we explored 18 graph structures and divided the GCN classification performance based on the population graph into three categories. Accurate measures with known links to AD pathologies substantially increase performance. Many tools for evaluating cognition and function in AD are available, but most of them lack the sensitivity necessary to detect MCI and disease progression. Several studies [37,38] cite the CDR-SB measures as a promising candidate for AD trials. A graph structure is optimal when clusters of patients and healthy subjects can be well separated. Not surprisingly, the best result was achieved when CDR-SB was applied to the edge-assigning function. Medium-level performance was achieved when MMSE, ADAS-Cog11, ADAS-Cog13, FAQ, or ADNI-MEM were applied to the edge-assigning functions. It is likely that some low-quality graphs (e.g., ADNI-EF, ADNI-LAN, or ADNI-VS) carry noisy information, which has a negative impact on the results. The edge-assigning function in a population graph can significantly affect classification accuracy.

The current study investigated the impact of feature importance and node interactions. It did not aim to obtain a superior model for AD diagnosis. However, when the GCN models were evaluated by comparing their accuracy metrics to those of other state-of-the-art models, the proposed model achieved promising performance for binary and multi-class classification, as shown in Table 6. It is important to note that the results may differ depending on the ADNI subjects as well as the machine learning models used. Additionally, it may be challenging to conduct a fair comparison due to the variations in the test samples. Compared to the state-of-the-art methods, the proposed method has the following three main advantages: First, the 3D DenseNet can encode a more comprehensive level of feature abstraction. Second, the GCN works as a feature extractor on the population graph structure to learn graph embedding. Third, the population graph is being constructed with different sources of similarities.

## 5. Conclusions

To evaluate how the input properties of a GCN affect AD staging performance, we applied a novel framework for the diagnosis of AD that integrated CNNs and GCNs into a unified network, and thereby took advantage of the outstanding feature expression of CNNs, and the good graph processing performance of GCNs. We performed three binaries: AD/CN, MCI/CN, and AD/MCI, and one multiclass AD/MCI/CN classification task. Experiments are implemented using data from ADNI-1. We achieved an accuracy of 91.6% on AD versus CN, 91.2% on AD versus MCI, 96.8% on MCI versus CN, and 89.4% on AD/MCI/CN classification tasks. Our method outperformed several other systems in the prior part. The promising performance was achieved by incorporating the following three factors: (1) The 3D DenseNet provides good feature abstractions. (2) The GNN provides a good graph embedding. (3) Rich complementary information was used in edge-assigning functions. Our findings confirmed our initial hypothesis that imaging features and pairwise information are crucial to the AD categorization process.

There were limitations to the proposed method. First, the population graph is a set of nodes connected by edges. In the ADNI-1 cohort, there were around 800 subjects; therefore, the population graph consisted of approximately 800 nodes. If thousands of subjects were contained in a graph, the topological structure of the graph would differ. Each node might be connected with too many neighbors, and the over-smoothing issue would be likely to occur; in this case, an edge sub-sampling strategy is required. Second, our graph encompasses several types of non-imaging information on the same edge. For example, age, gender, and ApoE were given the same weight when a composite score was calculated. An interesting extension would be to learn the weight of non-imaging information on edge-assigning function during training. This would allow for the gathering of complementary information and would weight the influence of some measures differently. Third, the GCN that we used was based on a simple layer-wise propagation rule. Applying imaging features in an edge-assigning function can be viewed as a kind of self-attention; edges to different nodes were modulated by their imaging features’ similarities. In some cases, the strategy of edge-assigning functions improved the model’s performance; in other cases, it degraded it. The graph attention network, which specifies different weights for different nodes in a neighborhood, may address the shortcomings of edge-assigning functions. Fourth, this study has used structural imaging features; however, adding functional imaging features could improve the model’s predictive ability, and future research could determine how to incorporate functional imaging features efficiently into a GNN architecture.

## Figures and Tables

**Table 1:** Demographic information and neuropsychological assessment results of the subjects in ADNI.

Characteristic	AD	MCI	CN	*p*-Values
Age	75.3 ± 7.5	74.7 ± 7.5	75.9 ± 5.0	*p* = 0.145
Gender (Male/Female)	98/89	245/137	119/110	<0.01 ^ABD^
ApoE4(0/1/2)	64/87/36	171/161/43	168/56/5	<0.01 ^ABCD^
MMSE	23.3 ± 2.0	27.3 ± 1.8	29.1 ± 1.0	<0.01 ^ABC^
CDR-SB	4.4 ± 1.6	1.6 ± 0.9	0.0 ± 0.1	<0.01 ^ABC^
ADAS-Cog11	19.7 ± 4.9	11.5 ± 4.4	6.2 ± 2.9	<0.01 ^ABC^
ADAS-Cog13	30.3 ± 6.1	18.7 ± 6.2	9.5 ± 4.2	<0.01 ^ABC^
FAQ	13.2 ± 6.8	3.8 ± 4.4	0.1 ± 0.6	<0.01 ^ABC^
ADNI-MEM	−0.9 ± 0.6	−0.1 ± 0.6	1.0 ± 0.5	<0.01 ^ABC^
ADNI-EF	−1.0 ± 0.9	−0.1 ± 0.9	0.6 ± 0.7	<0.01 ^ABC^
ADNI-LAN	−0.8 ± 0.9	−0.1 ± 0.8	0.8 ± 0.7	<0.01 ^ABC^
ADNI-VS	−0.6 ± 0.9	−0.1 ± 0.8	0.2 ± 0.6	<0.01 ^ABC^

A: significant differences (*p* < 0.05) between AD and MCI; B: significant differences (*p* < 0.05) between MCI and CN; C: significant differences (*p* < 0.05) between AD and CN; D: The χ2 test was used.

**Table 2:** GCN model performance under AD versus CN task on the test data with different edge-assigning functions. The best results are given in bold.

Edge-Assigning Function	ACC (%)	PRE (%)	REC (%)	F1 (%)	MCC (%)
$S_{img}$	86.8	84.2	86.5	85.3	71.3
$S_{\mathrm{com}}$ (age)	85.5	83.8	83.8	83.8	68.2
$S_{\mathrm{com}}$ (gender)	86.8	84.2	86.5	85.3	71.3
$S_{\mathrm{com}}$ (ApoE)	88.0	82.9	91.9	87.2	74.8
$S_{\mathrm{com}}$ (age and gender)	86.8	84.2	86.5	85.3	71.3
$S_{\mathrm{com}}$ (age and ApoE)	90.4	89.2	89.2	89.2	78.7
$S_{\mathrm{com}}$ (gender and ApoE)	89.2	88.9	86.5	87.7	75.8
$S_{\mathrm{com}}$ (age, gender and ApoE)	**91.6**	**91.7**	**89.2**	**90.4**	**81.1**

**Table 3:** The optimal threshold *β* of each neuropsychological assessment.

Classification Task	MMSE	CDR-SB	ADAS-Cog11	ADAS-Cog13	FAQ	ADNI-MEM	ADNI-EF	ADNI-LAN	ADNI-VS
AD versus MCI	1	2	3	3	5	0.3	0.3	0.7	0.5
MCI versus CN	2	1.5	5	3	1	1	1	0.3	0.3

**Table 4:** GCN model performance under AD versus MCI task on the test data with different edge-assigning functions. The best results are given in bold.

Edge-Assigning Function	ACC, PRE, REC, F1, MCC (%)		
	$S_{img}$	$S_{nimg}$	$S_{com}$
$S_{img}$	66.7, 49.0, 54.0, 51.4, 26.2		
$S_{nimg}\;\mathrm{or}\;S_{\mathrm{com}}$ (MMSE)		83.3, 100, 48.7, 65.5, 62.3	85.1, 95.5, 56.8, 71.2, 65.8
$S_{nimg}\;\mathrm{or}\;S_{\mathrm{com}}$ (CDR-SB)		**91.2, 90.9, 81.1, 85.7, 79.6**	**87.7, 89.7, 70.0, 79.1, 71.3**
$S_{nimg}\;\mathrm{or}\;S_{\mathrm{com}}$ (ADAS-Cog11)		79.0, 78.3, 48.7, 60.0, 49.3	79.8, 75.0, 56.8, 64.6, 51.8
$S_{nimg}\;\mathrm{or}\;S_{\mathrm{com}}$ (ADAS-Cog13)		80.7, 82.6, 51.4, 63.3, 53.8	80.7, 72.7, 64.9, 68.6, 54.9
$S_{nimg}\;\mathrm{or}\;S_{\mathrm{com}}$ (FAQ)		83.3, 75.0, 73.0, 74.0, 61.7	85.1, 79.4, 73.0, 76.1, 65.4
$S_{nimg}\;\mathrm{or}\;S_{\mathrm{com}}$ (ADNI-MEM)		78.9, 78.3, 48.7, 60.0, 49.1	79.8, 71.9, 62.2, 66.7, 52.5
$S_{nimg}\;\mathrm{or}\;S_{\mathrm{com}}$ (ADNI-EF)		74.6, 70.0, 37.8, 49.1, 37.1	74.6, 72.2, 35.1, 47.3, 36.9
$S_{nimg}\;\mathrm{or}\;S_{\mathrm{com}}$ (ADNI-LAN)		70.2, 60.0, 24.3, 34.6, 23.0	74.6, 64.3, 48.7, 55.4, 38.9
$S_{nimg}\;\mathrm{or}\;S_{\mathrm{com}}$ (ADNI-VS)		67.5, 50.0, 5.0, 9.80, 6.0	73.7, 63.0, 46.0, 53.1, 36.3

**Table 5:** GCN model performance under MCI versus CN task on the test data with different edge-assigning functions. The best results are given in bold.

Edge-Assigning Function	ACC, PRE, REC, F1, MCC (%)		
	$S_{img}\;$	$S_{nimg}$	$S_{com}$
$S_{img}$	68.3, 74.4, 75.3, 74.8, 37.7		
$S_{nimg}\;\mathrm{or}\;S_{\mathrm{com}}$ (MMSE)		74.8, 73.0, 94.8, 82.5, 56.4	78.1, 82.9, 81.8,82.3, 55.0
$S_{nimg}\;\mathrm{or}\;S_{\mathrm{com}}$ (CDR-SB)		**96.8, 95.1, 100, 97.5, 93.1**	**96.8, 97.0, 84.4, 90.3, 93.6**
$S_{nimg}\;\mathrm{or}\;S_{\mathrm{com}\;}$ (ADAS-Cog11)		77.2, 80.3, 84.4, 82.3, 54.7	78.9, 82.3, 84.4, 83.3, 57.3
$S_{nimg}\;\mathrm{or}\;S_{\mathrm{com}}$ (ADAS-Cog13)		85.4, 89.3, 87.0, 88.1, 68.9	83.7, 84.8, 87.0, 85.9, 66.0
$S_{nimg}\;\mathrm{or}\;S_{\mathrm{com}}$ (FAQ)		82.9, 98.3, 74.0, 84.4, 61.1	82.1, 98.3, 72.7, 83.6, 59.2
$S_{nimg}\;\mathrm{or}\;S_{\mathrm{com}}$ (ADNI-MEM)		75.0, 74.0, 94.0, 82.8, 56.2	86.2, 88.5, 89.6, 89.0, 71.0
$S_{nimg}\;\mathrm{or}\;S_{\mathrm{com}}$ (ADNI-EF)		67.5, 66.7, 96.1, 78.7, 48.2	67.5, 66.7, 96.1, 78.7, 48.2
$S_{nimg}\;\mathrm{or}\;S_{\mathrm{com}}$ (ADNI-LAN)		69.9, 73.8, 80.5, 77.0, 42.4	69.9, 73.8, 80.5, 77.0, 42.4
$S_{nimg}\;\mathrm{or}\;S_{\mathrm{com}}$ (ADNI-VS)		62.6, 62.6, 100, 77.0, 45.5	62.6, 62.6, 100, 77.0, 45.5

**Table 6:** Comparison of the proposed models with the state-of-the-art models.

Study	Model	Dataset	Image Modality	ACC (%)		
				AD versus CN	AD versus MCI	MCI versus CN
Kang et al. 2021 [5]	2D DCGAN	AD: 187 MCI: 382 CN: 229	T1-weighted MRI	90.36	77.16	72.36
Liu et al. 2020 [6]	3D UNet + DenseNet	AD: 97 MCI: 233 CN: 119	T1-weighted MRI	88.90		76.20
Tufail et al. 2022 [39]	3D VGG	AD: 94 MCI: 97 CN: 102	PET	86.63	68.50	62.22
Jiang et al. 2020 [11]	HI-GCN	AD: 34 MCI: 99	rs-fMRI		78.50	
An et al. 2020 [40]	GCN	AD: 78 CN: 145	rs-fMRI	91.30		
Li et al. 2021 [41]	TE-HI-GCN	AD: 34 MCI: 99	rs-fMRI		89.40	
Li et al. 2022 [42]	RBF-GCN	AD: 169 MCI: 165 CN: 168	T1-weighted MRI; DWI; amyloid-PET	96.06	92.73	95.15
Proposed method	3D DenseNet + GCN	AD: 187 MCI: 382 CN: 229	T1-weighted MRI	91.6	91.2	96.8

## Data Availability

The dataset is owned by a third-party organization; the Alzheimer’s Disease Neuroimaging Initiative (ADNI). Data are publicly and freely available from the http://adni.loni.usc.edu/data-samples/access-data/ (accessed on 20 December 2022), Institutional Data Access/Ethics Committee (contact via http://adni.loni.usc.edu/data-samples/access-data/, accessed on 20 December 2022) upon sending a request that includes the proposed analysis and the named lead.
